# Effect of Oxygen Extraction (Brush-Sign) on Baseline Core Infarct Depends on Collaterals (HIR)

**DOI:** 10.3389/fneur.2020.618765

**Published:** 2021-01-06

**Authors:** Adrien Guenego, Matthew Leipzig, Robert Fahed, Eric S. Sussman, Tobias D. Faizy, Blake W. Martin, David G. Marcellus, Max Wintermark, Jean-Marc Olivot, Gregory W. Albers, Maarten G. Lansberg, Jeremy J. Heit

**Affiliations:** ^1^Interventional and Diagnostic Neuroradiology, Stanford Medical Center, Palo Alto, CA, United States; ^2^Division of Neurology, Department of Medicine, Ottawa Hospital, Ottawa, ON, Canada; ^3^Toulouse Stroke Center, Toulouse, France; ^4^Stanford Stroke Center, Stanford University School of Medicine, Stanford, CA, United States

**Keywords:** stroke, interventional, MRI perfusion imaging, MRI susceptibility weighted imaging, thrombectomy

## Abstract

**Objectives:** Baseline-core-infarct volume is a critical factor in patient selection and outcome in acute ischemic stroke (AIS) before mechanical thrombectomy (MT). We determined whether oxygen extraction efficiency and arterial collaterals, two different physiologic components of the cerebral ischemic cascade, interacted to modulate baseline-core-infarct volume in patients with AIS-LVO undergoing MT triage.

**Methods:** Between January 2015 and March 2018, consecutive patients with an AIS and M1 occlusion considered for MT with a baseline MRI and perfusion-imaging were included. Variables such as baseline-core-infarct volume [mL], arterial collaterals (HIR: TMax > 10 s volume/TMax > 6 s), high oxygen extraction (HOE, presence of the brush-sign on T2^*^) were assessed. A linear-regression was used to test the interaction of HOE and HIR with baseline-core-infarct volume, after including potential confounding variables.

**Results:** We included 103 patients. Median age was 70 (58–78), and 63% were female. Median baseline-core-infarct volume was 32 ml (IQR 8–74.5). Seventy six patients (74%) had HOE. In a multivariate analysis both favorable HIR collaterals (*p* = 0.02) and HOE (*p* = 0.038) were associated with lower baseline-core-infarct volume. However, HOE significantly interacted with HIR (*p* = 0.01) to predict baseline-core-infarct volume, favorable collaterals (low HIR) with HOE was associated with small baseline-core-infarct whereas patients with poor collaterals (high HIR) and HOE had large baseline-core-infarct.

**Conclusion:** While HOE under effective collateral blood-flow has the lowest baseline-core-infarct volume of all patients, the protective effect of HOE reverses under poor collateral blood-flow and may be a maladaptive response to ischemic stroke as measured by core infarctions in AIS-LVO patients undergoing MT triage.

## Introduction

Mechanical thrombectomy (MT) is an effective treatment for acute ischemic stroke due to large-vessel occlusion (AIS-LVO) (1–5). MT eligible patients have a relatively small baseline-core-infarct volume at the time of imaging evaluation, and patients with favorable arterial collaterals are more likely to present with a small core infarction (6, 7) and to have less core infarction growth (8). Up to 40% of AIS-LVO patients may experience rapid early core infarct expansion, which often renders patients ineligible for MT at the time of imaging evaluation (9). Therefore, imaging biomarkers that would help to a better understanding of the physiologic response to ischemic challenge or that are predictive of core infarct growth would be beneficial in AIS patients undergoing MT triage.

The hypoperfusion intensity ratio (HIR) is derived from computed tomography (CT) or magnetic resonance (MR) perfusion imaging and has emerged as a powerful imaging predictor of favorable collaterals, decreased core infarction growth, and favorable clinical outcomes (8, 10–12). HIR is defined as the ratio of the brain tissue volume with a Time-to-Maximum delay (TMax, in seconds) > 10 s divided by that with a TMax > 6 s, such that a lower HIR ratio indicates a less severe perfusion delay and more robust collaterals (10, 13). Therefore, a low HIR (<0.4 or <0.5 according to studies) is a favorable imaging biomarker of MT eligibility (12) and outcome after treatment (14, 15).

Although cerebral collateral assessment by HIR or CT angiogram techniques provide important information to determine MT treatment eligibility, these techniques do not provide a complete understanding of the brain's physiologic response to ischemic challenge. For example, how well ischemic brain tissue extracts oxygen may influence core infarction size, penumbral volumes, and patient outcome. High oxygen extraction (HOE) in AIS-LVO patients may be measured by the presence of prominent hypointense signal within the medullary veins within ischemic tissue, which has been termed the Brush sign (16, 17). However, the relationship of these variables, especially the degree of oxygen extraction, to cerebral arterial collaterals is not clearly established in humans with AIS-LVO.

We hypothesized that the degree of oxygen extraction interacts with the ability of arterial collaterals to maintain small core infarct volumes and prevent core infarct growth in AIS-LVO patients. We determined whether HOE (Brush sign presence) interacted with arterial collaterals (HIR) to modulate baseline core infarction volume in patients with AIS-LVO undergoing MT triage.

## Methods

The study protocol was approved by the institutional review board and complied with the Health Insurance Portability and Accountability Act (HIPAA). Patient informed consent was waived by our review board for this single center retrospective analysis of anonymized data acquired prospectively. Adherence to the STROBE criteria (18) was enforced.

### Population and Clinical Data

Between January 2015 and March 2018, consecutive AIS-LVO patients with a M1 occlusion who underwent MT triage at our comprehensive stroke center were retrospectively reviewed in this single-center study. Included patients underwent MT triage by MRI that included an axial T2^*^ sequence, MR angiogram and MR perfusion. Patient clinical and treatment data were extracted from a prospectively maintained stroke database and the electronic medical record.

### Imaging Data and Evaluation

All imaging were performed on either a 1.5T GE Signa or 3.0T GE MR750 MRI scanner using standard departmental protocols, using an eight channel GE HR brain coil (GE Healthcare, Milwaukee, Wisconsin). Technical details and parameters for the sequences used in this study were as follows.

Diffusion Weighted Imaging parameters: TR = 6,000 ms, TE = 78.2 ms; *b*-value = 0 and 1,000 s/mm^2^; flip angle 90°, and slice thickness of 5 mm.

Perfusion-weighted imaging was performed using a dynamic susceptibility contrast technique following the intravenous administration of Multihance (529 mg/ml; Bracco, Milan, Italy) at a dose of 0.2 ml/kg body weight into an antecubital vein at a rate of 4.0 mL/s using a power injector (19). Perfusion parameters were: TR = 1,800 ms, TE = 35 ms; flip angle 80°, and slice thickness of 5 mm. Standard perfusion maps, including time-to-maximum of the residue function (TMax), were generated using the automated RAPID software (iSchemaView, Menlo Park, CA) (20). Quantitative perfusion delays were assessed as the volumes of cerebral tissue with TMax delays of >6 and 10 s using RAPID.

The ischemic penumbra was defined as the volume of brain tissue with a TMax >6 s on gadolinium-based dynamic-contrast susceptibility imaging.

Collaterals were measured by HIR, which was defined as the volume of brain tissue with TMax >10 s volume divided by the volume of tissue with TMax >6 s (10). A lower HIR ratio indicates a less severe perfusion delay and more robust collaterals (8, 10, 12, 13).

Core infarction and TMax volume measurements were quantified by an automatic software (RAPID, iSchemaView, Menlo Park, CA, USA). The core-penumbra mismatch volume and ratio was determined by a comparison of core infarction and penumbra volumes (21).

T2^*^ gradient-echo axial sequences were mostly performed as: TR 650.0 ms, TE 15.0 ms, slice was 5 mm, slice gap of 0.0, FOV of 24.0 × 24.0. Brush-sign was defined as an asymmetric hypo-intense area along the course of the sub-ependymal and medullary veins in the deep white matter (16, 17) and was assessed as present or absent on baseline T2^*^ imaging by two neuro-radiologists, XX. and XX., with respectively 4 and 7 years of practice. Disagreements were resolved by consensus readings.

### Outcomes and Statistical Analyses

Primary outcome was the interaction of brush-sign on T2^*^ and collaterals as defined by their HIR with baseline-core-infarct volume on Diffusion Weighted Imaging.

A descriptive analysis of the data was performed. Nominal variables were summarized using frequency descriptive analysis then compared using Fisher's exact test. Continuous variables were treated using mean, standard deviation, 95% confidence interval (IC95), median, quartiles and inter-quartile ranges, then tested on univariate analysis using the Mann-Whitney test. Normality of the variables was tested by the Shapiro-Wilk test.

A linear regression was used to analyze the interaction of brush-sign with the full scale of HIR values and their interaction to predict baseline-core-infarct volume in these patients. We also included potential confounding variables in the model based on published literature, such as age, presentation National Institute of Health Stroke Scale (NIHSS), onset to imaging time, and admission blood glucose. Initial agreement between the two interventional neuroradiologists was measured using Kappa of Cohen, then disagreements were resolved by consensus reading.

All statistical analyses were performed with R version 3.6.2 (22). *P*-value <0.05 was set for significance.

### Data Availability Statement

The datasets used and analyzed during the current study will be made available by the corresponding author upon reasonable request.

## Results

A total of 103 patients were included (see Flow-Chart in Supplementary Figure 1). Median patient age was 70 (58–78) years, baseline NIHSS was 17 (12–22), 65/103 patients (63%) were female, and 60 (58.3%) received intravenous thrombolysis. Patient clinical and imaging baseline characteristics are presented in Tables 1, 2.

**Table 1:** Baseline demographic and clinical data in the population, association with baseline-core-infarct volume.

	**All (*n* = 103)**	***p*-value**
Age, median (IQR)	70 (58–78)	0.04
Female, *n* (%)	65 (63%)	0.07
Mean Systolic Blood Pressure, mmHg (SD)	142 (32)	0.63
Median LDL (mg/dL, IQR)	105 (72–125)	0.18
Median Hemoglobin A1c% (IQR)	5.7 (5.4–6.5)	0.13
Median Admission Blood Glucose (mg/dL, IQR)	135 (111–180)	<0.0001
**Medical history**		
Hypertension, *n* (%)	77 (77%)	0.04
Diabetes, *n* (%)	33 (35%)	0.03
Hyperlipidemia, *n* (%)	52 (54%)	0.13
Atrial Fibrillation, *n* (%)	48 (52%)	0.4
Coronary Artery Disease, *n* (%)	22 (24%)	0.63
Prior Stroke, *n* (%)	11 (11.6%)	0.12
**Smoking**		
Never, *n* (%)	55 (56.7%)	0.85
Prior, *n* (%)	26 (26.8%)	
Current, *n* (%)	16 (16.5%)	
**Stroke details**		
Median time since LSN to imaging, min (IQR)	334 (238–430)	0.02
Median NIHSS (IQR)	17 (12–22)	0.0009
Intravenous Thrombolysis, *n* (%)	60 (58%)	0.78

*SD, standard deviation; IQR, interquartile range; CAD, coronary artery disease; LSN, last seen normal; NIHSS, National Institute of Health stroke scale*.

**Table 2:** Imaging characteristics in the population, association with baseline-core-infarct volume.

	**All (*n* = 103)**	***p*-value**
**Imaging characteristics**		
Baseline infarct core volume, ml, median (IQR)	32 (8–74.5)	
HOE (Brush sign presence) *n* (%)	76 (74%)	0.18
TMax 6 s volume, ml median (IQR)	39 (19.5–74)	<0.0001
TMax 10 s volume, ml median (IQR)	100 (64.3–132)	<0.0001
HIR median (IQR)	0.43 (0.27–0.625)	<0.0001
Mismatch volume, ml median (IQR)	54.9 (20.25–81)	<0.0001
Mismatch ratio median (IQR)	3 (1.4–9.95)	0.0007
1.5 Tesla MRI, *n* (%)	58 (56%)	0.38
**Vessel occlusion**		
MCA–M1, *n* (%)	103 (100%)	1
**Vessel occlusion side**		
Left, *n* (%)	56 (54%)	0.07

*SD, standard deviation; ICA, internal carotid artery; MCA, middle cerebral artery; M1, first segment of MCA; HOE, high oxygen extraction; TMax, Time-to-Maximum (seconds)*.

Median baseline-core-infarct volume was 32 mL (IQR, 8–74.5) and HIR was 0.43 (0.27–0.625). HOE, which was indicated by the Brush-sign, was present in 76 patients (74%), reader agreement was substantial (Cohen's Kappa 0.731) (23).

**Figure 1:**
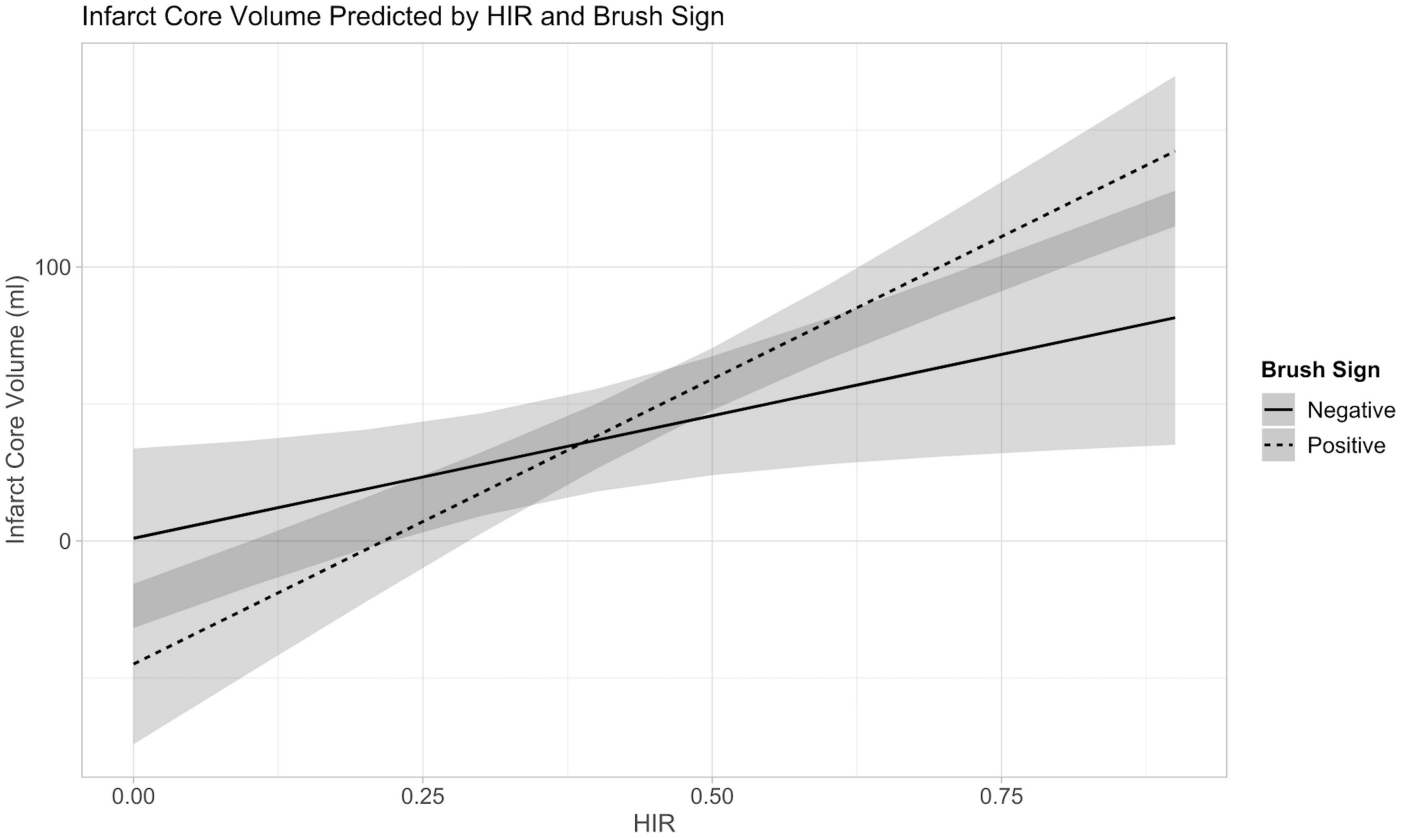
Interaction between Collateral Flow (HIR) and Oxygen Extraction (Brush-Sign) with Baseline-Core-Infarct Volume.

**Figure 2:**
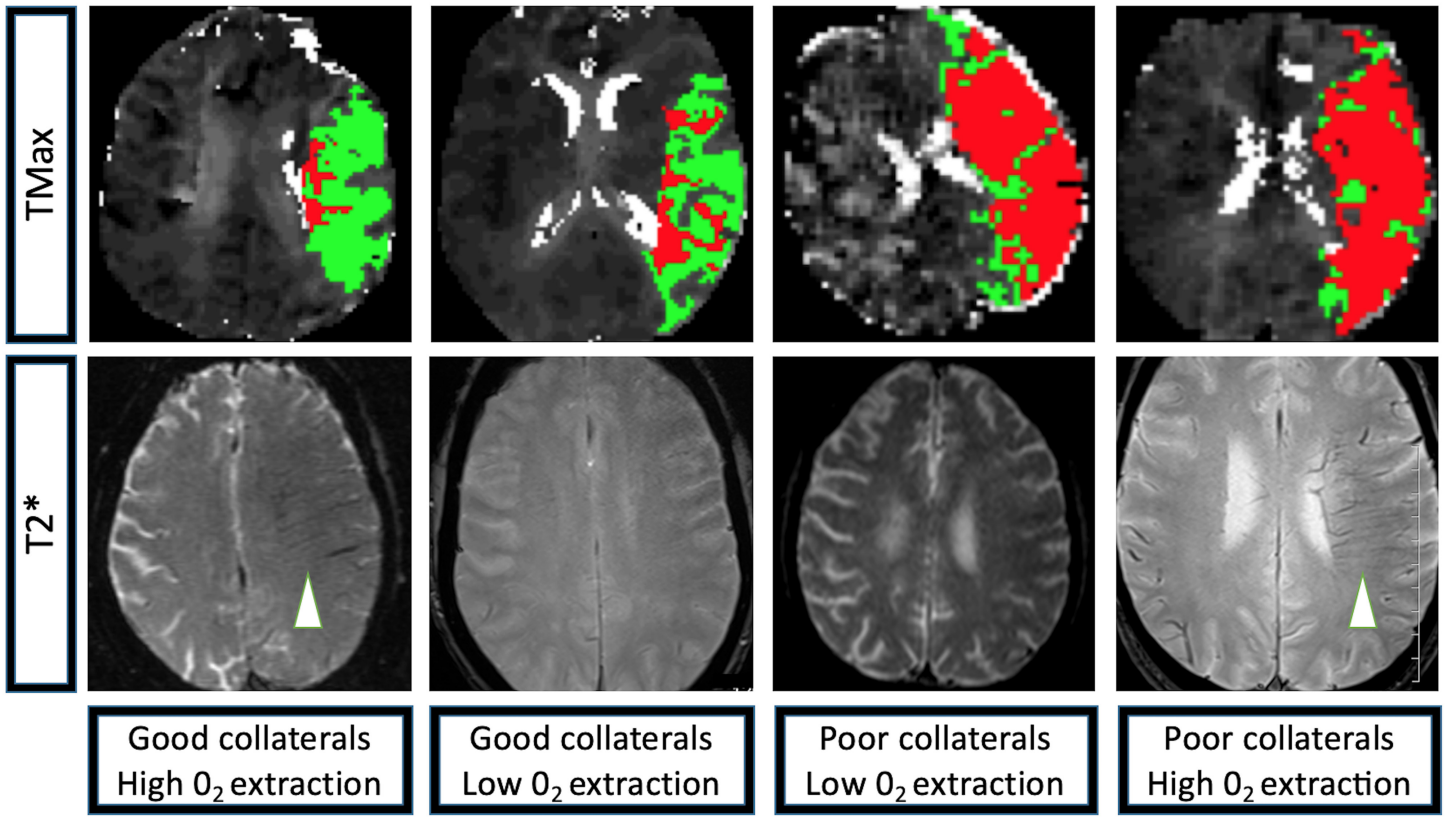
Perfusion and T2* characteristics of the four extreme imaging profiles with a left M1 occlusion. Group 1: Left M1 occlusion, HIR≤0.40 (good collaterals, low volume of tissue with TMax > 10 s [depicted in red] compared to the volume of tissue with TMax > 6 s [depicted in green]), high 0_2_ extraction (brush-sign on T2*, white arrowhead); Group 2: Left M1 occlusion, HIR≤0.40 (good collaterals), low 0_2_ extraction (no brush-sign on T2*); Group 3: Left M1 occlusion, HIR > 0.40 (poor collaterals, high volume of tissue with TMax>10 s [depicted in red] compared to the volume of tissue with TMax > 6 s [depicted in green]), low 0_2_ extraction (no brush-sign on T2*); Group 4: Left M1 occlusion, HIR > 0.40 (poor collaterals), high 0_2_ extraction (brush-sign on T2*, white arrowhead). TMax: Axial images of a magnetic resonance perfusion sequence depicting in red the volume of tissue with a TMax>10 s and in green the volume of tissue with TMax > 6 s. T2*: Axial images of a magnetic resonance GRE sequence, brush-sign is defined as an asymmetric hypo-intense area along the course of the sub-ependymal and medullary veins in the deep white matter and is outlined by a white circle.

The linear regression model of HIR, HOE, their interaction, age, presentation NIHSS, onset to imaging delay, and admission blood glucose as confounding variables revealed not only significant effects of HOE (*p* = 0.038) and HIR (*p* = 0.026), but also a significant association between HIR and HOE to predict baseline-core-infarct volume (*p* = 0.017) (Figure 1, Table 3). In patients with favorable collaterals (lower HIR), poor oxygen extraction (absent HOE [brush sign]) was associated with larger infarct core volumes compared to patients with HOE (brush sign present). By contrast, in patients with unfavorable collaterals (high HIR), HOE (brush sign present) was associated with larger infarct core volumes compared to patients with poor oxygen extraction (no HOE [brush sign]) (examples in Figure 2).

**Table 3:** Multivariate model for interactions with baseline-core-infarct volume.

	**Core volume (ml)**		
**Predictors**	**Estimates**	**CI**	***p***
(Intercept)	33.17	−35.14–101.48	0.337
HOE (Brush Sign)	−45.92	−89.25 – −2.58	**0.038**
HIR	89.47	10.95–168.00	**0.026**
Age	−0.96	−1.63 – −0.30	**0.005**
Presentation NIHSS	0.48	−1.10–2.05	0.550
Onset imaging	0.05	0.00–0.09	**0.039**
Admission blood glucose	0.08	−0.07–0.22	0.287
HOE (Brush sign) * HIR	118.55	21.67–215.44	**0.017**
Observations	103

*Bold values are p-values with a statistical significance <0.05*.

## Discussion

In this study of AIS-LVO patients undergoing MT triage, we found that HOE in patients with favorable collaterals is associated with small core infarctions at the time of presentation. By contrast, HOE in patients with poor collaterals was associated with larger core infarctions. Interestingly, HOE was not significantly associated with baseline-core-infarct volume in univariate but in multivariate analysis, these findings indicate the oxygen extraction and collateral blood flow are important modulators of brain tissue preservation during ischemic challenge due to AIS-LVO.

AIS-LVO results in the sudden disruption of blood flow to the brain, which must adapt to this ischemic challenge by maximizing blood flow to the brain to prevent irreversible core infarction. Patients with favorable collaterals are able to deliver blood to the ischemic brain and minimize core infarction growth. Patients who are able to maximally extract oxygen from the blood delivered by favorable collaterals are likely to further protect against core infarction growth due to increased oxygen delivery to ischemic brain tissue. Our findings support this hypothesis and interpretation of the ischemic cascade. Moreover, our findings suggest that neuroprotective agents that augment oxygen extraction might be beneficial in preventing core infarction growth in patients with robust collaterals, which has implications for AIS-LVO patients undergoing transfer from a primary stroke center to a comprehensive stroke center, where MT may be performed.

Our finding that HOE (brush sign presence) in patients with poor collaterals correlates with large core infarction volumes suggests that even a maximal physiologic response to ischemia with high levels of oxygen extraction are unable to prevent core infarction growth. Thus, the ability of collaterals to deliver blood to ischemic tissue may be more important for the preservation of brain tissue than effective oxygen extraction. This finding is consistent with prior MT trials that have found collaterals to be an important predictor of core infarction volume and outcome after MT (8, 12, 15, 24–27). The identification of neuroprotective agents that augment collateral blood flow are therefore of paramount importance for these patients.

Our results might also explain discrepant findings in prior studies that have evaluated oxygen extraction in the context of AIS. One study found that HOE in AIS patients is associated with larger baseline core infarctions and a greater risk of hemorrhagic transformation (16). However, the interaction between oxygen extraction and collateral robustness was not examined, which likely explains why increased oxygen extraction was correlated with larger baseline core infarctions (16) and poor clinical outcomes after intravenous-thrombolysis and MT treatment (28, 29).

By contrast, Derdeyn and colleagues showed that increased oxygen extraction in patients with cervical carotid artery occlusion may occur in the absence of cerebral blood volume elevation (a marker of collateral blood flow) (30). Furthermore, patients who were maximally adapted to augment blood flow to an ischemic hemisphere (increased oxygen extraction and cerebral blood volume) were at an increased risk for subsequent cerebral infarction (30). These findings are similar to our study, both of which indicate that oxygen extraction and cerebral perfusion due to collaterals might be uncoupled depending upon an individual patient's physiology and ability to respond to ischemic challenge. Our results and those of Derdeyn and colleagues also suggest that patients with poor collaterals and high rates of oxygen extraction are at the highest risk for core infarction and core infarction growth, as brain tissue damage related to oxidative stress may be maximized.

### Limitations

Our study is limited by its retrospective and single center design, which may introduce bias and limit the generalizability of our findings. We did not include patients without perfusion imaging, with CT imaging or with SWI imaging which may further limit the generalizability of our findings. The use of both 1.5 and 3.0 Tesla MRIs in our study might affect the detection of the brush-sign due to differences in technique, which may also introduce bias in our study.

## Conclusion

Robust collateral blood flow and high oxygen extraction are associated with small core infarctions in AIS-LVO patients undergoing MT triage. However, in patients with poor collaterals, increased oxygen extraction does not protect against core infarction growth prior to presentation. Maximal oxygen extraction and cerebral perfusion are variables that may be uncoupled depending upon an individual patient's physiologic adaptation to cerebral ischemia.

## Data Availability Statement

The raw data supporting the conclusions of this article will be made available by the authors, upon reasonable request.

## Ethics Statement

The studies involving human participants were reviewed and approved by Stanford Ethics committee. Written informed consent for participation was not required for this study in accordance with the national legislation and the institutional requirements.

## Author Contributions

AG, ML, RF, ES, TF, BM, DM, MW, J-MO, GA, MGL, and JH contributed to study conception and design, data collection, analysis and interpretation of results, and manuscript preparation. ML conducted all the statistical analyses. All authors contributed to the article and approved the submitted version.

## Conflict of Interest

The authors declare that the research was conducted in the absence of any commercial or financial relationships that could be construed as a potential conflict of interest.

## Supplementary Material

Supplementary Figure 1 Patients flow-chart. **352** (186 patients without an M1 occlusion) → **166** (61 patients had a CT as baseline imaging) → **105** (two patients had major artifacts on the baseline T2^*^ sequence, or the T2^*^ was not available) → **103** patients included.

## Abbreviations

AIS-LVO: Acute Ischemic Stroke due to Large-Vessel Occlusion
CT: Computed Tomography
HIR: Hypoperfusion Intensity Ratio
HOE: High Oxygen Extraction
M1: Proximal Middle Cerebral Artery
MR: Magnetic Resonance imaging
MT: Mechanical Thrombectomy
NIHSS: National Institute of Health Stroke Scale
TMax: Time-to-Maximum (seconds).
