# Supplementary figures and images for: Effect of Oxygen Extraction (Brush-Sign) on Baseline Core Infarct Depends on Collaterals (HIR)
Source: Front Neurol. 2021 Jan 6;11:618765. doi: 10.3389/fneur.2020.618765 (PMC7815586; doi:10.3389/fneur.2020.618765)

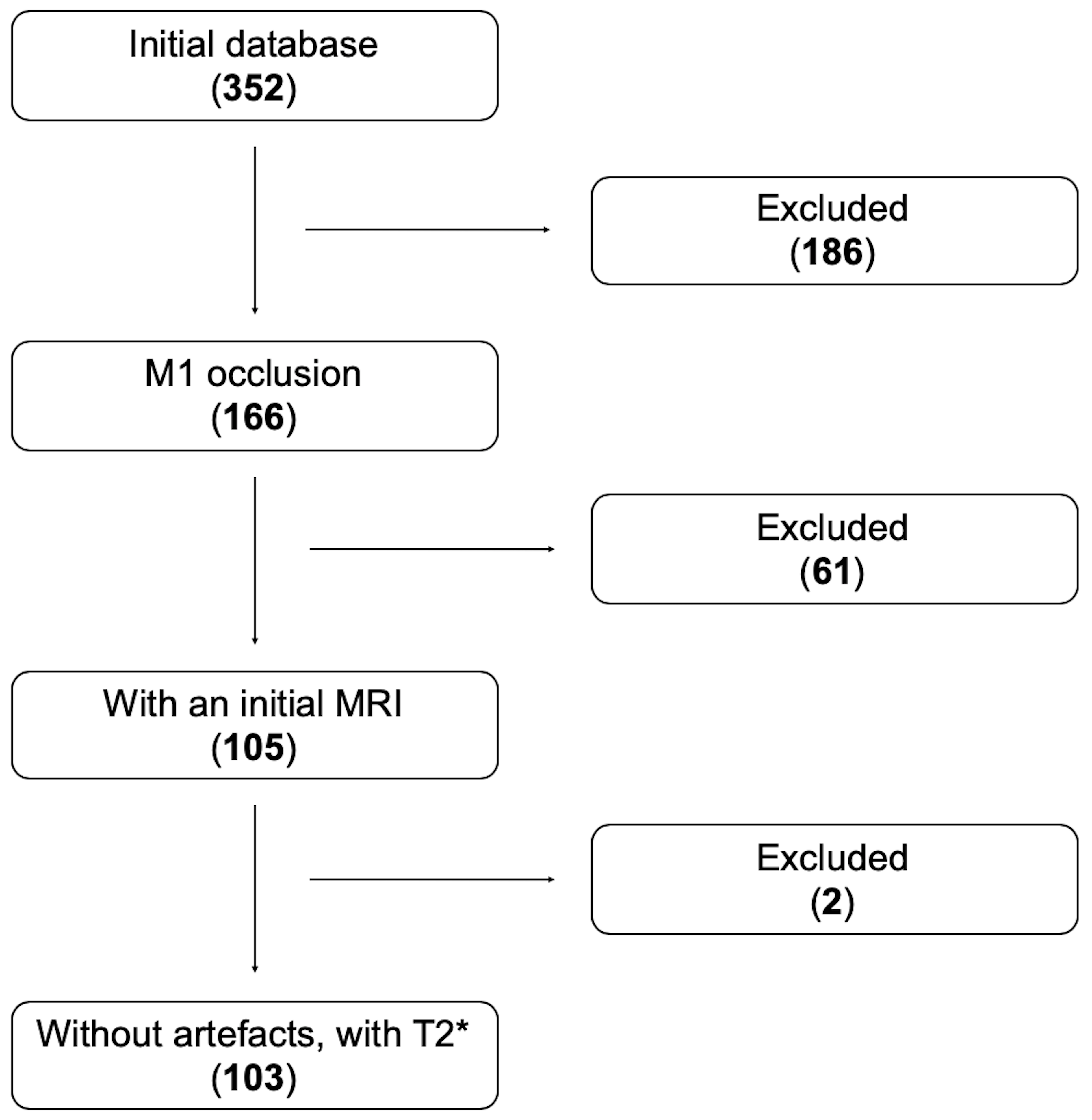

Supplement: Supplementary Figure 1 — Patients flow-chart. 352 (186 patients without an M1 occlusion) → 166 (61 patients had a CT as baseline imaging) → 105 (two patients had major artifacts on the baseline T2* sequence, or the T2* was not available) → 103 patients included. [file Image_1.TIFF]
